# Functional expression of bradykinin B_1_ and B_2_ receptors in neonatal rat trigeminal ganglion neurons

**DOI:** 10.3389/fncel.2015.00229

**Published:** 2015-06-15

**Authors:** Aya Kawaguchi, Masaki Sato, Maki Kimura, Takaki Yamazaki, Hitoshi Yamamoto, Masakazu Tazaki, Tatsuya Ichinohe, Yoshiyuki Shibukawa

**Affiliations:** ^1^Department of Dental Anesthesiology, Tokyo Dental CollegeTokyo, Japan; ^2^Department of Physiology, Tokyo Dental CollegeTokyo, Japan; ^3^Department of Histology and Developmental Biology, Tokyo Dental CollegeTokyo, Japan

**Keywords:** bradykinin, B_1_ receptor, B_2_ receptor, neuropathic pain, pain, trigeminal ganglion neuron, Ca^2+^ signaling

## Abstract

Bradykinin (BK) and its receptors (B_1_ and B_2_ receptors) play important roles in inflammatory nociception. However, the patterns of expression and physiological/pathological functions of B_1_ and B_2_ receptors in trigeminal ganglion (TG) neurons remain to be fully elucidated. We investigated the functional expression of BK receptors in rat TG neurons. We observed intense immunoreactivity of B_2_ receptors in TG neurons, while B_1_ receptors showed weak immunoreactivity. Expression of the B_2_ receptor colocalized with immunoreactivities against the pan-neuronal marker, neurofilament H, substance P, isolectin B4, and tropomyosin receptor kinase A antibodies. Both in the presence and absence of extracellular Ca^2+^ ([Ca^2+^]_o_), BK application increased the concentration of intracellular free Ca^2+^ ([Ca^2+^]_i_). The amplitudes of BK-induced [Ca^2+^]_i_ increase in the absence of [Ca^2+^]_o_ were significantly smaller than those in the presence of Ca^2+^. In the absence of [Ca^2+^]_o_, BK-induced [Ca^2+^]_i_ increases were sensitive to B_2_ receptor antagonists, but not to a B_1_ receptor antagonist. However, B_1_ receptor agonist, Lys-[Des-Arg^9^]BK, transiently increased [Ca^2+^]_i_ in primary cultured TG neurons, and these increases were sensitive to a B_1_ receptor antagonist in the presence of [Ca^2+^]_o_. These results indicated that B_2_ receptors were constitutively expressed and their activation induced the mobilization of [Ca^2+^]_i_ from intracellular stores with partial Ca^2+^ influx by BK. Although constitutive B_1_ receptor expression could not be clearly observed immunohistochemically in the TG cryosection, cultured TG neurons functionally expressed B_1_ receptors, suggesting that both B_1_ and B_2_ receptors involve pathological and physiological nociceptive functions.

## Introduction

Tissue damage results in an accumulation of endogenous chemical substances, such as bradykinin (BK), which are released by nociceptive afferents and/or non-neural cells in the injured area of the tissue (Julius and Basbaum, 2001; Basbaum et al., 2009). BK receptors, which are divided into two subtypes (B_1_ and B_2_), are plasma membrane G-protein-coupled receptors of the seven-transmembrane-domain family. The existence of B_1_ and B_2_ receptors has been confirmed by pharmacological and radioligand-binding studies, as well as by mRNA expression analyses, in a wide variety of cells (Hess et al., 1994; Pesquero et al., 1996; Hall, 1997). Previous studies have indicated that B_2_ receptors couple with the Gq protein. Activation of the Gq protein activates phospholipase C, which induces a number of intracellular second messenger systems, including 1, 2-diacylglycerol and inositol 1, 4, 5-trisphosphate, which activates protein kinase C and mobilizes intracellular Ca^2+^, respectively (Walker et al., 1995; Tiwari et al., 2005).

BK-induced changes in the chemical environment surrounding axons cause peripheral sensitization, which is associated with inflammatory responses (Basbaum et al., 2009). Neuropathic pain is also involved in peripheral and central sensitization, which increases chronic pain states (Cervero and Laird, 1996; Scholz and Woolf, 2002; Ochoa, 2009). Injury to trigeminal ganglion (TG) neurons, which occasionally induces neuropathic pain, has been reported to be mediated by both B_1_ and B_2_ receptors in the orofacial area. Formalin-induced orofacial pain responses in rats are reduced by B_2_ receptor inhibition (Chichorro et al., 2004). In addition, administration of B_1_ and B_2_ receptor antagonists delays the development of thermal hyperalgesia in the orofacial area, which is induced by constriction of the infraorbital nerve in rats and mice (Luiz et al., 2010). Thus, the functional role of BK receptors in TG neurons in physiological and pathological nociception has been well described by behavioral studies. However, the basic expression patterns of B_1_ and B_2_ receptors in TG neurons are still unclear and remain to be fully elucidated.

In the present study, we investigated the expression and localization, as well as physiological and pharmacological properties, of B_1_ and B_2_ receptors in primary cultured rat TG neurons.

## Materials and Methods

### Ethical Approval

All the animals used in our study were treated in accordance with the Guiding Principles for the Care and Use of Animals in the Field of Physiological Sciences, which was approved by the Council of the Physiological Society of Japan and the American Physiological Society. In addition, the study followed the guidelines that were established by the National Institutes of Health (USA) regarding the care and use of animals for experimental procedures. This study was approved by the Animal Research Ethics Committee of Tokyo Dental College (approval No. 252502).

### Cell Culture

TG cells were isolated from neonatal Wistar rats (7 days old) (Kawaguchi et al., 2015) that were under pentobarbital sodium anesthesia (50 mg/kg) following the administration of isoflurane (3.0 Vol%). TG cells were dissociated by enzymatic treatment with Hank’s balanced salt solution (Life Technologies, Grand Island, NY, USA) containing 20 U/mL papain (Worthington Biochemical Corporation, Lakewood, NJ, USA) for 20 min at 37°C, which was followed by dissociation by trituration. After dissociation, the TG cells were plated on 35 mm-diameter dishes (Corning Incorporated Life Sciences, Tewksbury, MA, USA) and cultured for 48 h at 37°C (95% air and 5% CO_2_). The primary cells were cultured in Leibovitz’s L-15 medium (Life Technologies) containing 10% fetal bovine serum, 1% penicillin-streptomycin (Life Technologies), 1% fungizone (Life Technologies), 26 mM NaHCO_3_, and 30 mM glucose (pH 7.4). For the immunocytochemistry, TG cells were subjected to primary culture on poly-L-lysine-coated cover glasses (Matsunami Glass Ind., Ltd., Osaka, Japan).

### Immunofluorescence Analysis

TGs isolated from neonatal Wistar rats (7 days old) were fixed in optimal cutting temperature compound and rapidly frozen in liquid nitrogen. Frozen tissues were cut at a thickness of 10 μm and placed on slides. After fixation by 50% ethanol and 50% acetone at −20°C for 30 min, primary cultured TG cells and cryosections were treated with 10% donkey serum at room temperature for 20 min and then incubated overnight at 4°C with primary antibodies (Kuroda et al., 2013). A cocktail of primary antibodies (Neuro-Chrom^TM^ Pan Neuronal Marker, EMD Millipore, Billerica, MA, USA; 1:50 dilution), including mouse anti-Neuronal nuclei (NeuN), anti-microtubule-associated protein 2 (MAP2), anti-βIII tubulin, and anti-neurofilament H (NF-H) antibodies, was used as a neuronal marker. TG cells were also incubated with either mouse anti-NF-H (SantaCruz, CA, USA; 1:200 dilution) as an A-neuron marker, mouse anti-substance P (SP; R&D Systems, Minneapolis, MN, USA; 2.5 μg/100 μl dilution) as a peptidergic C-neuron marker, FITC-conjugated anti-isolectin B4 (IB4; Vector laboratories, CA, USA; 1:200 dilution) as a non-peptidergic C-neuron marker, goat anti-high-affinity nerve growth factor (NGF) receptor (a tropomyosin receptor kinase A (TrkA); R&D Systems; 1.5 μg/100 μl dilution) as an NGF-responsive nociceptor marker (Mantyh et al., 2011), and rabbit anti-B_1_ receptor (Alomone Labs, Jerusalem, Israel; 1:50 dilution) and rabbit anti-B_2_ receptor (Alomone Labs; 1:50 dilution) (Duehrkop et al., 2013; Dutra et al., 2013) antibodies. For negative controls, the sections were incubated with non-immune IgGs (Abcam, Cambridge, UK; 1:50; *N* = 4 from four rats) (Figure 2M). The cells and tissues were then washed and incubated with a secondary antibody at room temperature for 30 min. The secondary antibodies were Alexa Fluor 488 donkey anti-rabbit IgG, Alexa Fluor 568 donkey anti-mouse IgG, Alexa Fluor 568 donkey anti-rabbit IgG, and Alexa Fluor 568 donkey anti-goat IgG (1:50 dilution; Life Technologies) for the fluorescence staining and 4′, 6-diamino 2-phenylindole dihydrochloride (Life Technologies) for the nuclear staining (room temperature for 5 min). The cells and tissues were examined under fluorescence microscopes (Carl Zeiss AG, Jena, Germany; Keyence Corporation, Osaka, Japan).

### Solutions and Reagents

A standard solution containing (in mM) 137 NaCl, 5.0 KCl, 2.0 CaCl_2_, 0.5 MgCl_2_, 0.44 KH_2_PO_4_, 0.34 Na_2_HPO_4_, 4.17 NaHCO_3_, and 5.55 glucose (pH 7.4) was used as an extracellular solution. A high-K^+^ solution containing (in mM) 91 NaCl, 50 KCl, 2.0 CaCl_2_, 0.5 MgCl_2_, 0.44 KH_2_PO_4_, 0.34 Na_2_HPO_4_, 4.17 NaHCO_3_, and 5.55 glucose (pH 7.4) was used to discern TG neurons from glial cells by activation of depolarization-induced increases in intracellular free Ca^2+^ concentration ([Ca^2+^]_i_) in neurons. BK, a selective B_2_ receptor antagonist (HOE140), a selective B_1_ receptor antagonist (R715) and a highly selective B_1_ receptor agonist (Lys-[Des-Arg^9^]BK) were obtained from Tocris Bioscience (Bristol, UK). All the other reagents were purchased from Sigma-Aldrich Co. LLC (St. Louis, MO, USA), except where indicated.

### Measurement of [Ca^2+^]_i_

Primary cultured TG cells were loaded for 90 min at 37°C in Hank’s solution containing 10 μM of fura-2 acetoxymethyl ester (Dojindo Laboratories, Kumamoto Japan) and 0.1% (w/v) pluronic acid F-127 (Life Technologies). Cultured TG cells were then rinsed with fresh Hank’s solution and mounted on a microscope stage (Olympus Corporation, Tokyo, Japan). Fura-2 fluorescence emission was measured at 510 nm in response to alternating excitation wavelengths of 340 nm (*F*340) and 380 nm (*F*380) with an Aquacosmos system and software (Hamamatsu Photonics K.K., Shizuoka, Japan), which controls the excitation wavelength selector and intensified charge-coupled device camera system (Hamamatsu Photonics K.K.). [Ca^2+^]_i_ was measured as the fluorescence ratio of *F*340 and *F*380 (R*_F340/F380_*) and expressed as *F/F*_0_ units. The R*_F340/F380_* value (*F*) was normalized to the resting value (*F*_0_).

### Statistical and Offline Analysis

The data were expressed as the mean ± standard error (S.E.) or standard deviation of the mean of *N* observations, where *N* represents the number of independent experiments or cells, respectively. The Kruskal–Wallis test, Dunn’s posthoc test, or Mann–Whitney *U*-test was used to determine the nonparametric statistical significance. *P* values less than 0.05 were considered significant. The statistical analysis was performed with GraphPad Prism 5.0 (GraphPad Software, Inc., La Jolla, CA, USA).

The dependence of the changes in [Ca^2+^]_i_ on each pharmacological agent was determined by fitting the data to the following function with Origin 8.5 (OriginLab Corporation, Northampton, MA, USA):

(1) $$F/F_{0}=\left[\left(F/F_{\text{0int}}-F/F_{0\text{fin}}\right)/\left(1+\left({\left[x\right]}_{\text{o}}/K\right)\right)\right]+F/F_{0\text{fin}}$$

where *K* is the equilibrium binding constant, [x]*_o_* indicates the applied concentration of the pharmacological agents, and *F/F*_0int_ and *F/F*_0fin_ are the initial and final *F/F*_0_ responses, respectively.

## Results

### Immunolocalization of BK Receptors in TG Neurons

**Figure 1:**
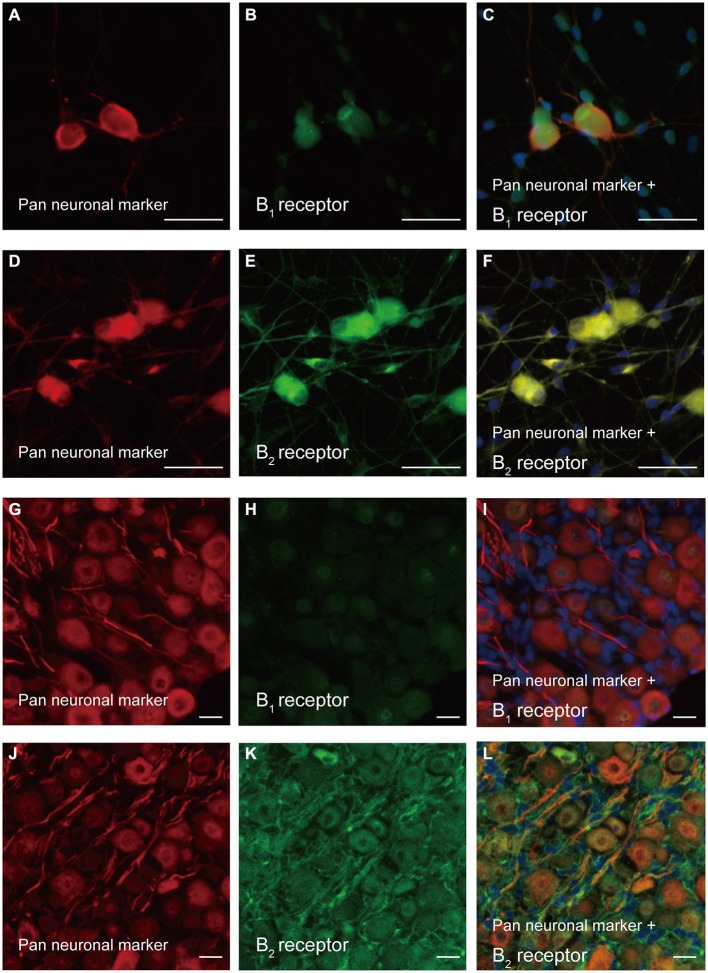
**Immunolocalization of B_1_ and B_2_ receptors in primary cultured trigeminal ganglion (TG) neurons and TG cryosections. (A,D,G,J)** Cells positive for the pan neuronal marker in primary cultured TG neurons **(A,D)** and TG cryosections **(G,J). (B,H)** Immunoreactivity to the B_1_ receptor antibody (green) in primary cultured TG neurons **(B)** and TG cryosections **(H). (C,I)** Triple immunofluorescence staining with antibodies against the pan neuronal marker (red) and B_1_ receptor (green) in primary cultured TG neurons **(C)** and TG cryosections **(I)**. Nuclei are shown in blue. **(E,K)** Positive immunoreactivity to the B_2_ receptor antibody (green) in primary cultured TG neurons **(E)** and TG cryosections **(K). (F,L)** Triple immunofluorescence staining with antibodies against the pan neuronal marker (red) and B_2_ receptor (green) in primary cultured TG neurons **(F)** and TG cryosections **(L)**. Nuclei are shown in blue. Scale bars are 50 μm in **(A–F)**, and 20 μm in **(G–L)**. Each set of images showing representative immunolocalization of B_1_ **(A–C)** and B_2_ receptors **(D–F)** in primary cultured TG neurons was obtained from six different rats, while that showing immunolocalization of B_1_ **(G–I)** and B_2_ receptors **(J–L)** in TG cryosections was obtained from five different rats.

The cultured TG neurons showed positive immunoreactivity to a neuronal marker cocktail (Neuro-Chrom^TM^ pan-neuronal marker), which contained mouse anti-NeuN, anti-MAP2, and anti-βIII tubulin antibodies (Figures 1A,D). Intense B_2_ receptor immunoreactivity was observed in primary cultured TG neurons (Figure 1E), and it showed colocalization with the pan neuronal marker (Figure 1F) in somata, dendrites, axons, and perinuclear regions. Weak but positive B_1_ receptor immunoreactivity was also observed in primary cultured TG cells (Figure 1B), and the immunoreactivity colocalized with the pan neuronal marker (Figure 1C).

**Figure 2:**
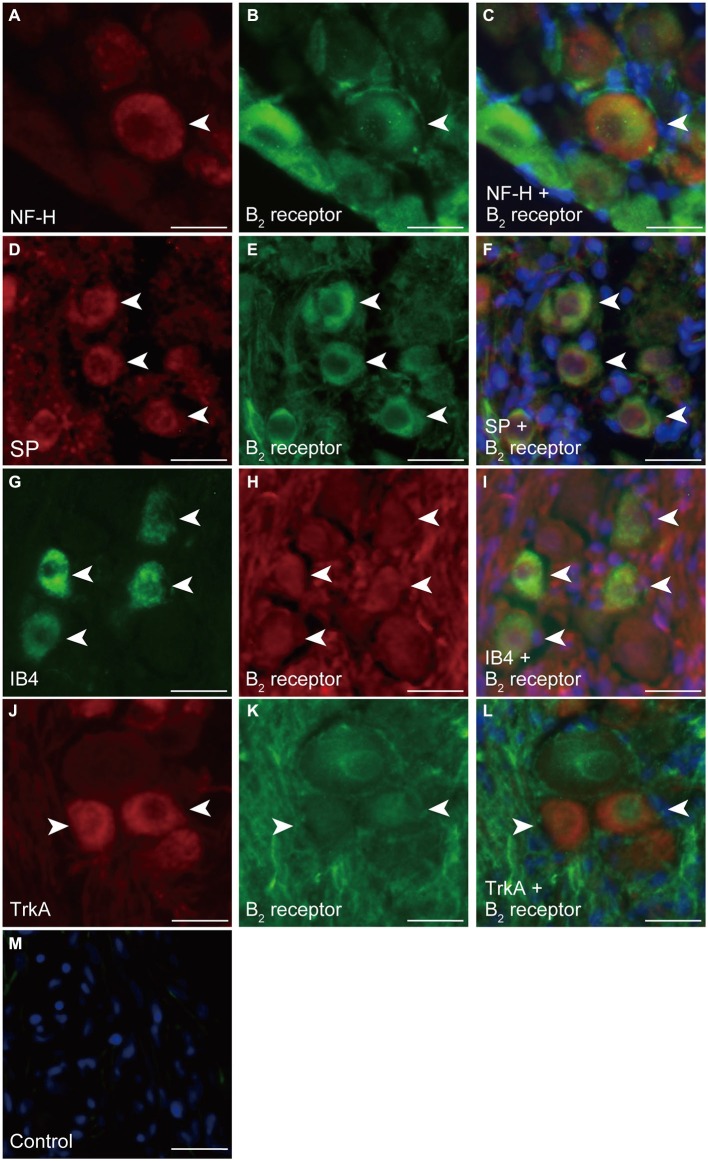
**Immunolocalization of B_2_ receptors in the soma of TG neurons in cryosections. (A)** Positive immunoreactivity to NF-H as an A-neuron marker in TG neurons (arrowhead). **(B,E,H,K)** B_2_ receptor immunoreactivity (arrowheads). **(C)** Triple immunofluorescence staining with antibodies against B_2_ receptors (green) and NF-H (red). Nuclei are shown in blue. **(D)** Positive immunoreactivity to SP as a peptidergic C-neuron marker in TG neurons (arrowheads). **(F)** Triple staining with antibodies against B_2_ receptors (green) and SP (red). Nuclei are shown in blue. **(G)** Positive immunoreactivity to IB4 as a non-peptidergic C-neuron marker in TG neurons (arrowheads). **(I)** Triple staining with antibodies against B_2_ receptors (red) and IB4 (green). Nuclei are shown in blue. **(J)** Positive immunoreactivity to TrkA as an nerve growth factor (NGF)-responsive nociceptor marker in TG neurons (arrowheads). **(L)** Triple staining with antibodies against B_2_ receptors (green) and TrkA (red). Nuclei are shown in blue. **(M)** No fluorescence was detected in the negative control. Scale bars: 20 μm. Each set of photos showing representative colocalization of B_2_ receptors with NF-H **(A–C)** and SP **(D–F)** was obtained from six different rats. Each set of photos showing representative colocalization of B_2_ receptors with IB4 **(G–I)** and TrkA **(J–L)** was obtained from four different rats.

In the TG cryosections, we could observe positive immunoreactivity against the neuronal marker cocktail (Figures 1G,J). These TG neurons in the cryosections showed positive immunoreactivity to the B_2_ receptor antibody (Figure 1K), showing colocalization with the pan neuronal marker (Figure 1L) in somata, dendrites, axons, and perinuclear regions. However, the TG cryosections did not show B_1_ receptor immunoreactivity (Figures 1H,I). Positive immunoreactivity was also observed with NF-H (an A-neuron marker; Figure 2A), SP (a peptidergic C-neuron marker; Figure 2D), IB4 (a non-peptidergic C-neuron marker; Figure 2G), and high-affinity NGF receptor (TrkA; an NGF-responsive nociceptor marker; Figure 2J) antibodies. These immunoreactivities against NF-H, SP, IB4, and TrkA antibodies showed colocalization with those against the B_2_ receptor antibodies (Figures 2B,C,E,F,H,I,K,L).

### BK-Induced [Ca^2+^]_i_ Increases in TG Neurons

**Figure 3:**
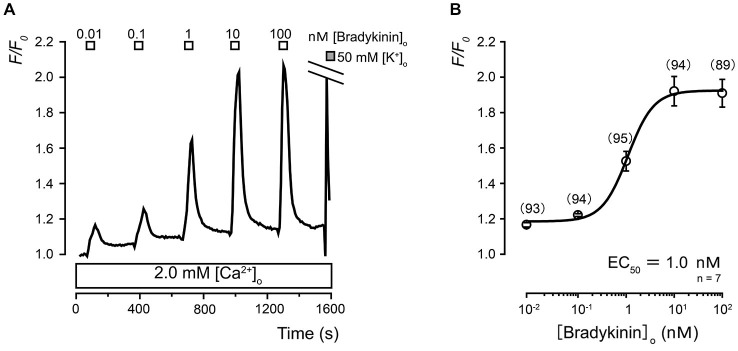
**Ca^2+^ dependence of extracellular bradykinin (BK)-induced [Ca^2+^]_i_ responses in TG neurons. (A)** Examples of transient [Ca^2+^]_i_ increases following the application of a series of BK concentrations. In the presence of extracellular Ca^2+^ (2.0 mM; lower white box), the application of BK induced transient [Ca^2+^]_i_ increases in a concentration-dependent manner. The concentrations of BK (0.01–100 nM) that were administered are shown in the uppermost white boxes. **(B)** The data points illustrate the *F/F*_0_ values as a function of the applied BK concentration. Each data point represents the mean ± standard error (S.E.) of seven experiments (the numbers in parentheses represent the number of tested cells). The curve on the semilogarithmic scale was fitted according to *Equation 1*, which is described in the text. The upper gray box in **(A)** indicates the timing of the application of the 50 mM KCl solution. The equilibrium binding constant of BK was 1.0 nM.

We observed rapid and transient [Ca^2+^]_i_ increases in TG neurons following the administration of five different concentrations of BK (0.01, 0.1, 1.0, 10, and 100 nM) in the presence of external Ca^2+^ (2.0 mM; Figure 3A). A semilogarithmic plot (Figure 3B) illustrates *F/F*_0_ values as a function of the applied BK concentrations, and the equilibrium-binding constant was the half-maximal 50% effective concentration (EC_50_) of 1.0 nM.

### HOE140, a B_2_ Receptor Antagonist, Inhibited the BK-Induced [Ca^2+^]_i_ Increases in TG Neurons

**Figure 4:**
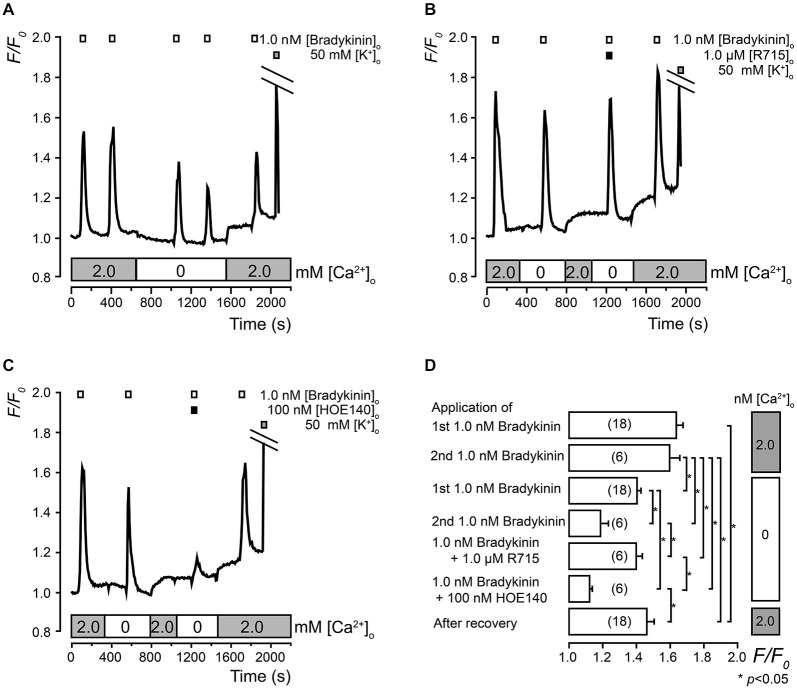
**Pharmacological identification of BK receptors in TG neurons. (A)** Examples of transient [Ca^2+^]_i_ increases following the administration of 1.0 nM of BK (upper white boxes) with (lower gray boxes) or without (lower white box) extracellular Ca^2+^ (2.0 mM). **(B)** Examples of BK-induced (1.0 nM; upper white boxes) [Ca^2+^]_i_ increases with (upper black box) or without R715 in the absence (lower white box) or presence (lower gray boxes) of external Ca^2+^. **(C)** Examples of BK-induced (1.0 nM; upper white boxes) [Ca^2+^]_i_ increases with (upper black box) or without HOE140 in the absence (lower white box) or presence (lower gray boxes) of external Ca^2+^. **(A,B,C)** The upper gray boxes indicate the timing of the application of the 50 mM of KCl solution. **(D)** The summary bar graph indicates [Ca^2+^]_i_ increases during the first (upper column) and second (second upper column) application of 1.0 nM of BK with external Ca^2+^ (2.0 mM) (gray boxes on the right side). The mean values of the increase in [Ca^2+^]_i_ following the first (third upper column) and second (fourth upper column) application of 1.0 nM of BK in the absence of external Ca^2+^ as well as the application of 1.0 nM of BK with 1.0 μM of R715 (fifth upper column) and 100 nM of HOE140 (sixth upper column) in the absence of external Ca^2+^ (white boxes on the right side) are shown. Each column denotes the mean ± S.E. of the indicated (in parentheses) number of experiments. The statistical significance between the columns (shown by solid lines) is indicated by asterisks: *p < 0.05.

We examined the BK-induced [Ca^2+^]_i_ responses in both the presence and absence of external Ca^2+^. The application of BK (1.0 nM) rapidly increased [Ca^2+^]_i_ to a peak *F/F*_0_ value of 1.7 ± 0.03 *F/F*_0_ units in the presence (2.0 mM) of external Ca^2+^ and 1.4 ± 0.03 *F/F*_0_ units in the absence (0 mM) of external Ca^2+^ (Figures 4A,D). The amplitudes of the BK-induced [Ca^2+^]_i_ increases significantly differed between those in the presence and absence of extracellular Ca^2+^. In the absence of extracellular Ca^2+^, BK (1.0 nM)-induced [Ca^2+^]_i_ increases were significantly inhibited by a B_2_ receptor antagonist (100 nM of HOE140) (Figures 4C,D) but not by a B_1_ receptor antagonist (1.0 μM of R715) (Figures 4B,D).

### The B_1_ Receptor Antagonist R715 did not Affect the BK-Induced [Ca^2+^]_i_ Increases

**Figure 5:**
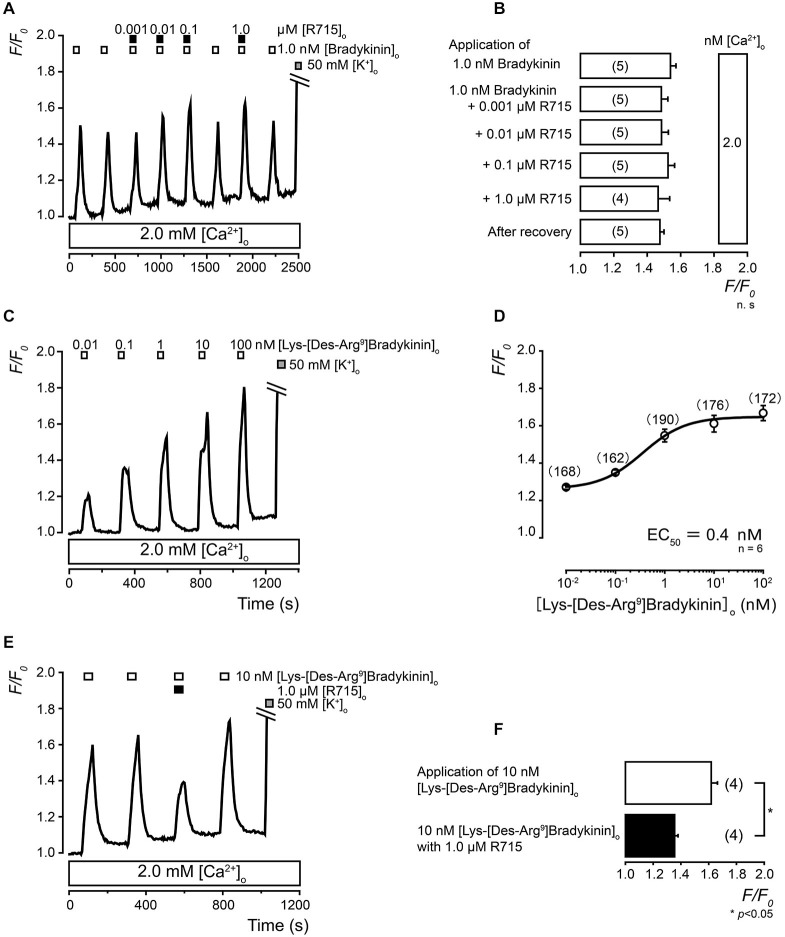
**Pharmacological identification of B_1_ receptors in TG neurons. (A)** Examples of BK-induced (1.0 nM; upper white boxes) [Ca^2+^]_i_ increases showing insensitivity to R715 (0.001–1.0 μM; upper black boxes) in the presence of external Ca^2+^ (2.0 mM; lower white box). **(B)** The summary bar graph indicates [Ca^2+^]_i_ increases following the first (upper column) application of 1.0 nM of BK with external Ca^2+^ (2.0 mM). The mean values for the increases in [Ca^2+^]_i_ following the application of 1.0 nM of BK with 0.001 μM (second upper column), 0.01 μM (third upper column), 0.1 μM (fourth upper column), or 1.0 μM (fifth upper column) of R715 in the presence of external Ca^2+^ (white boxes on the right side) are shown. Each column denotes the mean ± S.E. of the indicated (in parentheses) number of experiments. There is no statistical significance between the columns. **(C)** Examples of transient [Ca^2+^]_i_ increases following the application of a series of concentrations of Lys-[Des-Arg^9^]BK (0.01–100 nM; upper white boxes) in the presence of extracellular Ca^2+^ (2.0 mM; lower white box). **(D)** The data points illustrate the *F/F*_0_ values as a function of the applied concentrations of Lys-[Des-Arg^9^]BK. Each data point represents the mean ± S.E. of six independent experiments (the numbers in parentheses represent the number of tested cells). The curve on the semilogarithmic scale was fitted according to *Equation 1*, which is described in the text. The equilibrium-binding constant for Lys-[Des-Arg^9^]BK was 0.4 nM. **(E)** Examples of Lys-[Des-Arg^9^]BK-induced (10 nM; upper white boxes) [Ca^2+^]_i_ increases that were significantly inhibited by 1.0 μM of R715 (upper black box) in the presence of external Ca^2+^ (2.0 mM; lower white box). **(A,C,E)** The application of 50 mM of the KCl solution is shown in the gray boxes. **(F)** Summary bar graph of the [Ca^2+^]_i_ increases following 10 nM of Lys-[Des-Arg^9^]BK with (lower black column) or without (upper white column) 1.0 μM of R715. Each column denotes the mean ± S.E. of the indicated number (in parentheses) of independent experiments. The statistical significance between the columns (shown by solid lines) is indicated by asterisks: *p < 0.05.

The BK-induced (1.0 nM) increases in [Ca^2+^]_i_ were not significantly inhibited in TG neurons by the administration of four different concentrations of the B_1_ receptor antagonist (0.001, 0.01, 0.1, and 1.0 μM of R715) in the presence (2.0 mM) of external Ca^2+^ (Figures 5A,B).

### Pharmacological Identification of B_1_ Receptors in TG Neurons

We investigated the [Ca^2+^]_i_ increases during the administration of Lys-[Des-Arg^9^]BK, which is an endogenous, potent, and highly selective B_1_ receptor agonist (Talbot et al., 2009; More et al., 2014). The increases in [Ca^2+^]_i_ in the TG neurons were induced by the administration of five different concentrations of Lys-[Des-Arg^9^]BK (0.01, 0.1, 1, 10, and 100 nM) in the presence of extracellular Ca^2+^ (2.0 mM) (Figure 5C). A semilogarithmic plot (Figure 5D) illustrates the *F/F*_0_ values as a function of the applied concentration of Lys-[Des-Arg^9^]BK with an equilibrium-binding constant of 0.4 nM. In the presence of extracellular Ca^2+^, the Lys-[Des-Arg^9^]BK-induced increase in [Ca^2+^]_i_ was significantly inhibited by a B_1_ receptor antagonist (1.0 μM of R715) (Figures 5E,F).

## Discussion

The present study demonstrated the functional expression of BK receptors (B_1_ and B_2_) in TG neurons. B_2_ receptors were present on axons and dendrites in A-neurons, non-peptidergic C-neurons, peptidergic C-neurons, and NGF-responsive nociceptors. While the localization pattern of the B_1_ receptor was not clear in the TG cryosections, weak immunoreactivity for B_1_ receptors was observed in the primary cultured TG neurons. The application of BK activated B_2_ receptors and Lys-[Des-Arg^9^]BK activated the B_1_ receptors. B_2_ receptor activation mobilized [Ca^2+^]_i_ by releasing Ca^2+^ from internal Ca^2+^ stores with partial Ca^2+^ influx from the extracellular medium.

B_2_ receptors, which are expressed ubiquitously and constitutively in healthy tissues, are essential in the early stages of general pain generation (Hall, 1992). The constitutive expression of B_2_ receptors in TG neurons has been studied by reverse transcription-polymerase chain reaction (RT-PCR) analyses (Ceruti et al., 2011) and immunocytochemical analyses in cultured TG neurons (Patwardhan et al., 2005). Although BK-induced [Ca^2+^]_i_ increases have also been reported in TG neurons (Ceruti et al., 2008, 2011), precise functional expression patterns of B_1_ and B_2_ receptors in TG neurons remained unclear. The results of the present study showing the functional expression and localization of B_2_ receptors in TG neurons were in line with the previous results. The results of this study were also in line with the pharmacological properties of BK, which is a potent and endogenous agonist for B_2_ receptors and not B_1_ receptors in the sympathetic neurons of the rat superior cervical ganglion (Babbedge et al., 1995) and in Chinese hamster ovary (CHO) cells stably expressing recombinant human B_1_ or B_2_ receptors (Simpson et al., 2000). Furthermore, BK has an affinity for B_2_ receptors that is 500 times that for B_1_ receptors (Simpson et al., 2000). Therefore, B_2_ receptors are histologically and functionally expressed, and endogenous BK preferentially activates B_2_ receptors in rat TG neurons.

The expression of the B_1_ receptor, which is induced as a result of tissue damage and inflammation, is involved in chronic inflammation or tissue injury (Hall, 1992). The observations of the constitutive B_1_ receptor expression in TG and dorsal root ganglion (DRG) neurons have been inconsistent. In DRG neurons, some immunohistochemical studies have reported constitutive B_1_ receptor expression (Ma et al., 2000; Wotherspoon and Winter, 2000). In contrast, other studies have described that B_1_ receptor activation-induced [Ca^2+^]_i_ responses could not be observed in DRG neurons (Brand et al., 2001). In TG neurons, an immunohistochemical study has shown the constitutive expression of B_1_ receptors (Ma et al., 2000). In contrast, RT-PCR analyses have demonstrated that B_1_ receptor mRNA was barely expressed in intact tissue, while it was weakly expressed in primary cultured TG neurons. In primary cultured TG neurons, the levels of expression of B_1_ receptor mRNA have been reported to depend on the length of the culture period (Ceruti et al., 2011). The present immunohistochemical and immunocytochemical results were similar to the previous RT-PCR results; B_1_ receptor immunoreactivity was weakly positive in cultured TG neurons and could not be detected in intact TG tissue. Although few report concerning B_1_ receptor-induced [Ca^2+^]_i_ response in TG neurons exist, in the [Ca^2+^]_i_ imaging in the present study, the B_1_ receptor agonist, Lys-[Des-Arg^9^]BK which is a metabolite of endogenous BK in peripheral tissues (Regoli et al., 2001), dose-dependently increased [Ca^2+^]_i_ in the presence of extracellular Ca^2+^, and this increase was suppressed by a B_1_ receptor-specific antagonist (Figures 5C–F). These results of B_1_ receptor expression in primary cultured TG neurons suggest that the expression of B_1_ receptors is induced in TG neurons by tissue damage and/or inflammation. However, further studies are required to evaluate the expression patterns of B_1_ receptors in native TG neurons.

BK-induced [Ca^2+^]_i_ increases were observed in both the presence and absence of extracellular Ca^2+^. However, the amplitudes of the [Ca^2+^]_i_ increases in the absence of extracellular Ca^2+^ were significantly smaller (84.9 ± 11.3%, *N* = 161) than those in the presence of Ca^2+^ (100%; Figures 4A,D). This indicated that the BK-induced [Ca^2+^]_i_ mobilization (by B_2_ receptor activation) was mainly composed of Ca^2+^ release from internal stores with partial Ca^2+^ influx from the extracellular medium. Notably, BK has been reported to activate voltage-dependent Ca^2+^ channels in rat submucosal plexus neurons (Avemary and Diener, 2010; Rehn et al., 2013), and transient receptor potential cation channel subfamily-V member-1 channels in rat DRG neurons (Ferreira et al., 2004; Mistry et al., 2014). However, BK-induced Ca^2+^ currents could not be recorded in TG neurons (Kitakoga and Kuba, 1993). Although further studies are needed to clarify which Ca^2+^ influx pathways contribute to the BK-induced Ca^2+^ influx in TG neurons, the present results clearly indicate that BK mobilizes [Ca^2+^]_i_ through both intracellular Ca^2+^ release and Ca^2+^ influx.

In addition, NGF-TrkA signaling plays important roles in not only the developmental processes of peptidergic nociceptive afferents, but also in the generation of acute and chronic pain state in adults. The signaling also up-regulates B_2_ receptor expression in peptidergic nociceptors (Mantyh et al., 2011). Thus, the results showing colocalization of B_2_ receptor and TrkA immunoreactivity in TG neurons strongly support reports describing that the B_2_ receptor mediates inflammatory/neuropathic pain induced by peripheral sensitization in the orofacial region (Chichorro et al., 2004; Luiz et al., 2010); however, the present results obtained from neonatal rat may not reflect the situation in adults.

In conclusion, B_2_ receptors were expressed constitutively, and their activation induced the mobilization of [Ca^2+^]_i_ by releasing Ca^2+^ from intracellular stores with partial Ca^2+^ influx. In contrast, B_1_ receptor expression was faint in cultured TG neurons and absent in neurons in TG cryosections, although a metabolite of endogenous BK elicited [Ca^2+^]_i_ increases. These results indicated that both BK and its metabolites activated [Ca^2+^]_i_ mobilization in TG neurons through B_2_ and B_1_ receptor activation, respectively.

## Conflict of Interest Statement

The authors declare that the research was conducted in the absence of any commercial or financial relationships that could be construed as a potential conflict of interest.
